# Relationship Between Bronchodilator Reversibility and Spirometry Response to Dupilumab in Type 2 High Uncontrolled Severe Asthma

**DOI:** 10.1111/cea.14634

**Published:** 2025-01-28

**Authors:** Robert Greig, Kirsten Stewart, Chris RuiWen Kuo, Rory Chan, Brian Lipworth

**Affiliations:** ^1^ Scottish Centre for Respiratory Research University of Dundee Dundee UK

**Keywords:** asthma, bronchodilator, dupilumab, reversibility, spirometry

**Summary**

- Dupilumab's lung function response varies more than salbutamol's bronchodilator response in type 2 asthmatic patients.
- The patient's response to salbutamol and dupilumab may be relatively disconnected.

To the editor,

Airway hyperresponsiveness is a key tenet of persistent asthma, along with the type 2 inflammatory phenotype in most patients, while bronchodilator reversibility (BDR) may be absent in patients with a preserved FEV_1_. Biologics act on downstream type 2 cytokine pathways inhibiting signalling of interleukins (IL) 4, 5 and 13 [1]. A retrospective study in severe asthma patients receiving anti‐IL‐5 or anti‐IL‐5Rα showed no difference in clinical outcomes when stratified by baseline BDR. Dupilumab is a monoclonal antibody which targets the alpha subunit of IL4 receptor which in turn inhibits IL4 and IL13 signalling, resulting in reduced exacerbations, improved symptom control and better lung function [2]. We wanted to evaluate the putative relationship between baseline BDR to salbutamol and the lung function response to dupilumab, expressed as either force expiratory volume in 1 s (FEV_1_) or forced mid‐expiratory flow (FEF_25‐75_).

Here we assessed patients with uncontrolled severe asthma (EudraCT 2021‐005593‐25) who had BDR measured at their initial screening visit. Then after a 4 week run‐in period patients also had lung function measured after receiving 12 weeks of dupilumab 300 mg given every 2 weeks. We assessed the spirometry response to either salbutamol or dupilumab calculated as % predicted changes from baseline, as well as relative % change from baseline (post–pre/pre), and absolute change (FEV_1_ in L and FEF_25‐75_ as L/s). To ensure an equal comparison, the pre‐salbutamol baseline at screening prior to run‐in was used for both assessments.

Spirometry (Micromedical, Chatham, UK) measurements were performed in triplicate according to the ERS guidelines [3]. 400 μg of salbutamol via a pressurised metered dose inhaler with an Aerochamber spacer device (Trudell Medical, London, Canada) was administered to all patients and after 20 min, the spirometry was repeated to assess BDR. Following the manufacturer's instructions and ERS guidelines, FeNO was obtained using NIOX Vero (NIOX, Oxford, UK) [4]. SPSS version 29 was used for statistical analysis. Paired students *t*‐tests were applied with an alpha error of 5% and Pearsons test was used to evaluate correlations. Nominal *p* values are quoted as either *p* < 0.05, < 0.01 or < 0.001 (two tailed).

The cohort consisted of 24 patients, 14 females, mean (SEM) age 52.3 (2.96); BMI 30.0 (1.23). The mean % predicted pre‐bronchodilator pulmonary function values and type 2 inflammation markers were: FEV_1_ 88.1% (3.5); FEF_25‐75_ 47.5% (3.0); FVC 105.8% (4.0) ACQ 3.0 (0.2); FeNO 68.0 ppb (8.9); eosinophils 510/μL (48), total IgE 204.7 kU/l (42.8).

There were significant improvements in FEV_1_ and FEF_25‐75_ in response to salbutamol. However, the response to dupilumab was significant for FEV_1_ but not FEF_25‐75_ (Table 1). There were no significant differences when comparing FEV_1_ or FEF_25‐75_ responses between salbutamol versus dupilumab (Table 1).

**TABLE 1:** Change in FEV_1_ and FEF_25‐75_ in response to either salbutamol (bronchodilator response: BDR) or to dupilumab (after 12 weeks) with differences in response between dupilumab versus salbutamol. For each variable changes are shown as absolute values, as relative % change or as % predicted change.

	Salbutamol (from baseline)			Dupilumab (from baseline)			Dupilumab versus salbutamol
	Mean	95% CI	*p*	Mean	95% CI	*p*	Mean (95% CI) for difference, *p*
FEV_1_ (L)	0.20	0.11, 0.28	< 0.001	0.26	0.04, 0.49	< 0.05	0.07, (−0.14, 0.27), 0.49
Relative % change in FEV_1_ (%)	7.51	4.31, 10.71	< 0.001	8.97	2.04, 15.91	< 0.05	1.46, (−4.99, 7.91), 0.64
% predicted FEV_1_	6.21	3.77, 8.64	< 0.001	6.92	1.38, 12.45	< 0.05	0.71, (−4.47, 5.89), 0.78
FEF_25‐75_ (L/s)	0.31	0.13, 0.49	0.001	0.41	−0.04, 0.87	0.075	0.10, (−0.29, 0.49), 0.60
Relative % change in FEF_25‐75_ (%)	18.8	8.73, 28.93	< 0.001	23.71	0.66, 46.76	< 0.05	4.88, (−15.23, 24.99), 0.62
% predicted FEF_25‐75_	8.00	3.57, 12.43	0.001	9.08	−0.98, 19.15	0.075	1.08, (−7.76, 9.93), 0.80

Correlations between salbutamol BDR and dupilumab responses were weak for FEV_1_ (0.43, *p* < 0.05) and FEF_25‐75_ (0.52, p < 0.05).

There were no significant differences between proportions of salbutamol versus dupilumab responders who exceeded the minimal important differences for severe asthma [5] for ≥ 150 mL improvement in FEV_1_ (50% vs. 46%) or ≥ 0.21 L/s in FEF_25‐75_ (42% vs. 50%). The mean post dupilumab % predicted values were 95.02 FEV_1_ for 56.6 for FEF_25‐75_.

Our data shows that in patients with uncontrolled severe asthma both salbutamol and dupilumab resulted in a statistically significant improvements in FEV_1_ with a similar magnitude of mean changes. However, wider confidence intervals were seen in response to dupilumab compared to salbutamol, particularly evident when expressed as % predicted change in FEV_1_.

When calculated as % predicted change, the confidence intervals were wider comparing FEF_25‐75_ to FEV_1_ responses to either dupilumab or salbutamol. In this regard, using % predicted change is likely to account for differences in baseline airway geometry as compared to using relative % change. The FEF_25‐75_ measures volume dependent small airways closure which is predictive of poor asthma control in terms of salbutamol use and requirement for oral corticosteroids [6]. However, FEF_25‐75_ is also more variable than FEV_1_ when assessing BDR in severe asthma patients [7]. Hence the significant effects of dupilumab on FEV_1_ but not FEF_25‐75_ may reflect the greater variability in the latter along with the constraints of a limited sample size.

The wider variance for the dupilumab response along with its weak correlation to salbutamol response, may reflect the specific effects of salbutamol on airway smooth muscle alone via β_2_‐adrenoceptors, while dupilumab acts on airway smooth muscle along with type 2 mucosal inflammation via IL‐4 and IL‐13 [8]. Dupilumab has also been shown to result in larger improvements in FEV_1_ in patients with higher biomarkers of type 2 inflammation including eosinophils and FeNO^2^. Ideally, we might have repeated salbutamol BDR before and after the run‐in period. However, patients had a mannitol bronchial challenge test performed after the run‐in prior to the first dose of dupilumab, it was not feasible to also repeat the salbutamol BDR. Notably in the phase 3 LIBERTY QUEST study, dupilumab peak improvements in FEV_1_ were observed by 6 weeks [2] in turn suggesting that the 12 weeks duration was sufficient in our study. In QUEST the mean change in FEV_1_ in response to dupilumab 300 mg in patients with eosinophils > 300/μL was 0.24 L (95% CI 0.16, 0.32) which was comparable to 0.26 L (95% CI 0.04, 0.49) in our type 2 high patients, despite patients in QUEST having a lower % predicted FEV_1_ baseline value.

In conclusion, the wider variance for dupilumab response may reflect effects on both airway smooth muscle and on endobronchial type 2 inflammation. Thus, it is conceivable that patients with limited salbutamol reversibility may still obtain appreciable improvements in lung function in response to dupilumab.

## Author Contributions

Robert Greig: Statistical analysis and writing. Kirsten Stewart: Data collection and review. Chris RuiWen Kuo: Trial design and submission, review. Rory Chan: Trial design and submission, review. Brian Lipworth: Trial design, data interpretation and analysis, writing.

## Ethics Statement

No. 07/02/2022, 21/WS/0151, West of Scotland REC 1, EudraCT 2021–005593‐25.

## Conflicts of Interest

Dr. Kuo reports personal fees from AstraZeneca, personal fees from Chiesi, and non‐ financial support from GSK outside the submitted work. Dr. Chan reports personal fees (talks) and support attending ERS from AstraZeneca, personal fees (consulting) from Vitalograph, and personal fees (talks) from Thorasys. Dr. Lipworth reports non‐financial support (equipment) from GSK; grants, personal fees (consulting, talks and advisory board), other support (attending ATS and ERS) and from AstraZeneca; personal fees (talks and consulting) from Sanofi, personal fees (consulting, talks and advisory board) from Circassia in relation to the submitted work; grants, personal fees (consulting, talks, advisory board), other support (attending ERS) from Teva, personal fees (talks and consulting), grants and other support (attending ERS and BTS) from Chiesi, personal fees (consulting) from Lupin, personal fees (consulting) from Glenmark, personal fees (consulting) from Dr. Reddy, personal fees (consulting) from Sandoz; grants, personal fees (consulting, talks, advisory board), other support (attending BTS) from Boehringer Ingelheim, grants and personal fees (advisory board and talks) from Mylan outside of the submitted work; and the son of BJL is presently an employee of AstraZeneca. The other authors declare no conflicts of interest.

## Data Availability

The data that support the findings of this study are available from the corresponding author upon reasonable request.
